# 2-(3,4-Di­fluoro­phen­yl)-1*H*-benzimidazole

**DOI:** 10.1107/S1600536813028559

**Published:** 2013-10-23

**Authors:** M. S. Krishnamurthy, Nikhath Fathima, H. Nagarajaiah, Noor Shahina Begum

**Affiliations:** a Department of Chemistry, Bangalore University, Bangalore 560 001, India

## Abstract

In the title mol­ecule, C_13_H_8_F_2_N_2_, the dihedral angle between the benzimidazole ring system and the di­fluoro-substituted benzene ring is 30.0 (1)°. In the crystal, mol­ecules are linked by N—H⋯N hydrogen bonds, forming chains along [010]. In addition, weak C—H⋯F hydrogen bonds connect chains into a two-dimensional network parallel to (001). A weak C—H⋯π inter­action is observed between an H atom of the benzimidazole ring sytem and the π system of the di­fluoro-substituted benzene ring.

## Related literature

For the therapeutic and medicinal properties of benzimidazole derivatives, see: Chimirri *et al.* (1991 ▶); Ishihara *et al.* (1994 ▶); Kubo *et al.* (1993 ▶). For related structures, see: Rashid *et al.* (2007 ▶); Jayamoorthy *et al.* (2012 ▶); Yoon *et al.* (2012 ▶); Fathima *et al.* (2013 ▶).
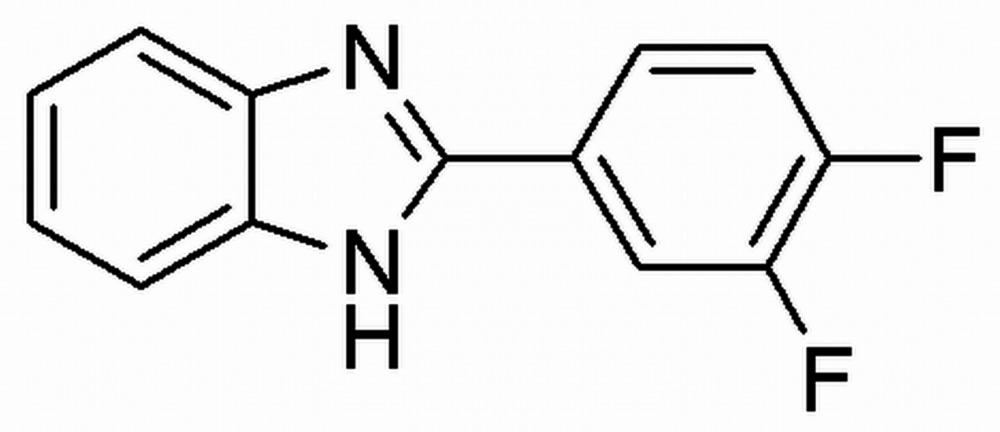


## Experimental

### 

#### Crystal data

C_13_H_8_F_2_N_2_
*M* *_r_* = 230.21
Orthorhombic, 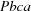

*a* = 8.7195 (17) Å
*b* = 9.9454 (19) Å
*c* = 23.389 (4) Å
*V* = 2028.2 (7) Å^3^
*Z* = 8
Mo *K*α radiation
μ = 0.12 mm^−1^
*T* = 100 K
0.18 × 0.16 × 0.16 mm

#### Data collection

Bruker SMART APEX CCD detector diffractometer
Absorption correction: multi-scan (*SADABS*; Bruker, 1998 ▶) *T*_min_ = 0.982, *T*_max_ = 0.984
13072 measured reflections
2209 independent reflections
1558 reflections with *I* > 2σ(*I*)
*R*_int_ = 0.067

#### Refinement

*R*[*F* ^2^ > 2σ(*F* ^2^)] = 0.059
*wR*(*F* ^2^) = 0.156
*S* = 1.01
2209 reflections
154 parameters
H-atom parameters constrained
Δρ_max_ = 0.47 e Å^−3^
Δρ_min_ = −0.31 e Å^−3^

### 

Data collection: *SMART* (Bruker, 1998 ▶); cell refinement: *SAINT-Plus* (Bruker, 1998 ▶); data reduction: *SAINT-Plus*; program(s) used to solve structure: *SHELXS97* (Sheldrick, 2008 ▶); program(s) used to refine structure: *SHELXL97* (Sheldrick, 2008 ▶); molecular graphics: *ORTEP-3 for Windows* (Farrugia, 2012 ▶) and *CAMERON* (Watkin *et al.*, 1996 ▶); software used to prepare material for publication: *WinGX* (Farrugia, 2012 ▶).

## Supplementary Material

Crystal structure: contains datablock(s) global, I. DOI: 10.1107/S1600536813028559/lh5659sup1.cif

Structure factors: contains datablock(s) I. DOI: 10.1107/S1600536813028559/lh5659Isup2.hkl

Supplementary material file. DOI: 10.1107/S1600536813028559/lh5659Isup3.cml

Additional supplementary materials:  crystallographic information; 3D view; checkCIF report

## Figures and Tables

**Table 1:** Hydrogen-bond geometry (Å, °) *Cg* is the centroid of the ring C9–C13 ring.

*D*—H⋯*A*	*D*—H	H⋯*A*	*D*⋯*A*	*D*—H⋯*A*
N1—H1⋯N2^i^	0.88	2.04	2.874 (3)	158
C13—H13⋯F2^ii^	0.95	2.51	3.379 (3)	153
C3—H3*A*⋯*Cg* ^iii^	0.95	2.89	3.529 (3)	125

Symmetry codes: (i) 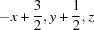; (ii) 
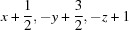
; (iii) 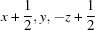.

**Fig. 1.**
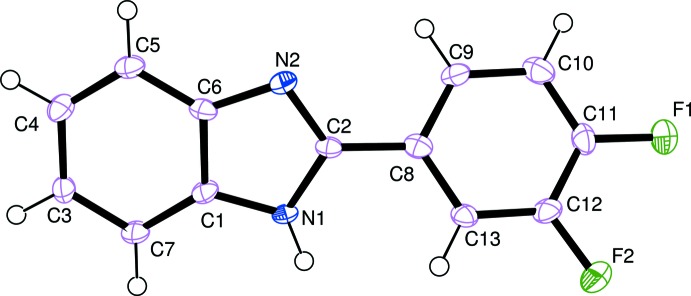
The molecular structure of the title compound with displacement ellipsoids drawn at the 50% probability level. H atoms are presented as small spheres of arbitrary radius.

**Fig. 2.**
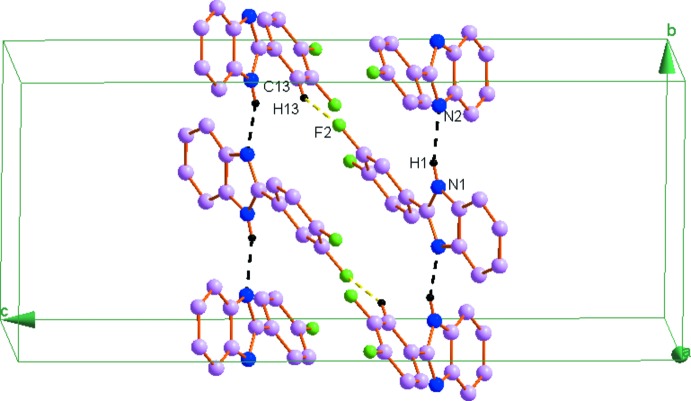
Part of the crystal structure showing intermolecular hydrogen bonds with dashed lines. H-atoms not involved in hydrogen bonds have been excluded. The atoms N2 and F2 are related by the symmetry operators (-x+3/2, y+1/2, z) and (x+1/2, -y+3/2, -z+1) respectively.

## supplementary crystallographic information

### 1. Comment

Benzimidazole is a bicyclic heterocycle system consisting of two nitrogen atoms and fused phenyl ring. It shows wide variety of pharmacological activities such as antihypertensive (Kubo *et al.*, 1993), anti-HIV (Chimirri *et al.*, 1991), antiulcer (Ishihara *et al.*, 1994). The bond lengths and bond angles of the benzimidazole moiety in the title compound are in good agreement with those observed in other benzimidazole derivatives (Jayamoorthy *et al.*, 2012; Yoon *et al.*, 2012).

The molecular structure of the title compound is shown in Fig. 1. The dihedral angle between the benzimidazole ring system and difluoro-substituted benzene ring is 30.0 (1)°. This value is slightly larger than for the benzene ring with a trifluoromethoxy substituent at the *para* position (Fathima *et al.*, 2013), and slightly smaller for a ring with a fluorine atom at the *para* position (Rashid *et al.*, 2007). In the crystal, molecules are linked by intermolecular N1—H1···N2^i^ and C9—H9···F2^ii^ hydrogen bonds (see Table 1 for symmetry codes). The former interaction forms extended chains parallel to the *b*-axis and the latter results in one-dimensional chains along the *a*-axis (Fig. 2). Overall a two-dimensional network parallel to (001) is formed. In addition, a weak C—H···π interaction of the type C3—H3A···*Cg* (*Cg* being the centroid of the ring C9—C13 ring) is observed (Table 1).

### 2. Experimental

The title compound was synthesized by refluxing 3,4-difluorobenzaldehyde (20 mmol,0.28 g) and *o*-phenyldiamine (20 mmol,0.22 g) in benzene (3.0 ml) for 6hrs on a water bath. The reaction mixture was cooled. The solid separated, was filtered and dried (Yield; 0.34 g (75%) and M.P. 533 K). Yellow crystals of the title compound were obtained by slow evaporation of a solution of the title compound in ethyl acetate.

### 3. Refinement

The H atoms were placed in calculated positions and refined in a riding-model approximation with C—H = 0.93 Å, N—H = 0.86 Å and with *U*_iso_(H) = 1.2*U*_eq_(N/C).

### Crystal data

**Table:**

C_13_H_8_F_2_N_2_	*F*(000) = 944
*M**_r_* = 230.21	*D*_x_ = 1.508 Mg m^−^^3^
Orthorhombic, *P**b**c**a*	Mo *K*α radiation, λ = 0.71073 Å
Hall symbol: -P 2ac 2ab	Cell parameters from 2209 reflections
*a* = 8.7195 (17) Å	θ = 2.9–27.0°
*b* = 9.9454 (19) Å	µ = 0.12 mm^−^^1^
*c* = 23.389 (4) Å	*T* = 100 K
*V* = 2028.2 (7) Å^3^	Block, yellow
*Z* = 8	0.18 × 0.16 × 0.16 mm

### Data collection

**Table:**

Bruker SMART APEX CCD detector diffractometer	2209 independent reflections
Radiation source: fine-focus sealed tube	1558 reflections with *I* > 2σ(*I*)
Graphite monochromator	*R*_int_ = 0.067
ω scans	θ_max_ = 27.0°, θ_min_ = 2.9°
Absorption correction: multi-scan (*SADABS*; Bruker, 1998)	*h* = −11→10
*T*_min_ = 0.982, *T*_max_ = 0.984	*k* = −11→12
13072 measured reflections	*l* = −28→29

### Refinement

**Table:**

Refinement on *F*^2^	Primary atom site location: structure-invariant direct methods
Least-squares matrix: full	Secondary atom site location: difference Fourier map
*R*[*F*^2^ > 2σ(*F*^2^)] = 0.059	Hydrogen site location: inferred from neighbouring sites
*wR*(*F*^2^) = 0.156	H-atom parameters constrained
*S* = 1.01	*w* = 1/[σ^2^(*F*_o_^2^) + (0.0725*P*)^2^ + 2.7172*P*] where *P* = (*F*_o_^2^ + 2*F*_c_^2^)/3
2209 reflections	(Δ/σ)_max_ < 0.001
154 parameters	Δρ_max_ = 0.47 e Å^−^^3^
0 restraints	Δρ_min_ = −0.31 e Å^−^^3^

### Special details

**Table:**

Geometry. All s.u.'s (except the s.u. in the dihedral angle between two l.s. planes) are estimated using the full covariance matrix. The cell s.u.'s are taken into account individually in the estimation of s.u.'s in distances, angles and torsion angles; correlations between s.u.'s in cell parameters are only used when they are defined by crystal symmetry. An approximate (isotropic) treatment of cell s.u.'s is used for estimating s.u.'s involving l.s. planes.
Refinement. Refinement of *F*^2^ against ALL reflections. The weighted *R*-factor *wR* and goodness of fit *S* are based on *F*^2^, conventional *R*-factors *R* are based on *F*, with *F* set to zero for negative *F*^2^. The threshold expression of *F*^2^ > 2σ(*F*^2^) is used only for calculating *R*-factors(gt) *etc*. and is not relevant to the choice of reflections for refinement. *R*-factors based on *F*^2^ are statistically about twice as large as those based on *F*, and *R*- factors based on ALL data will be even larger.

### Fractional atomic coordinates and isotropic or equivalent isotropic displacement parameters (Å^2^)

**Table:**

	*x*	*y*	*z*	*U*_iso_*/*U*_eq_	
F1	0.14852 (18)	0.58743 (18)	0.48006 (8)	0.0366 (5)	
F2	0.38138 (19)	0.75233 (17)	0.50344 (7)	0.0341 (5)	
N1	0.8188 (2)	0.5958 (2)	0.36510 (9)	0.0174 (5)	
H1	0.8081	0.6819	0.3727	0.021*	
N2	0.7666 (2)	0.3748 (2)	0.36264 (9)	0.0182 (5)	
C1	0.9394 (3)	0.5363 (2)	0.33662 (11)	0.0177 (5)	
C2	0.7191 (3)	0.4952 (2)	0.37920 (10)	0.0160 (5)	
C3	1.1638 (3)	0.4987 (3)	0.28151 (11)	0.0211 (6)	
H3A	1.2526	0.5311	0.2624	0.025*	
C4	1.1307 (3)	0.3607 (3)	0.28051 (11)	0.0219 (6)	
H4	1.1975	0.3017	0.2605	0.026*	
C5	1.0038 (3)	0.3082 (3)	0.30778 (11)	0.0204 (6)	
H5	0.9838	0.2143	0.3076	0.025*	
C6	0.9064 (3)	0.3973 (2)	0.33543 (11)	0.0175 (5)	
C7	1.0692 (3)	0.5888 (3)	0.30993 (11)	0.0212 (6)	
H7	1.0918	0.6822	0.3111	0.025*	
C8	0.5694 (3)	0.5218 (2)	0.40631 (11)	0.0185 (5)	
C9	0.4478 (3)	0.4362 (3)	0.39494 (12)	0.0228 (6)	
H9	0.4632	0.3615	0.3703	0.027*	
C10	0.3033 (3)	0.4573 (3)	0.41895 (13)	0.0268 (6)	
H10	0.2201	0.3988	0.4106	0.032*	
C11	0.2849 (3)	0.5654 (3)	0.45506 (12)	0.0233 (6)	
C12	0.4062 (3)	0.6501 (3)	0.46667 (12)	0.0216 (6)	
C13	0.5477 (3)	0.6304 (2)	0.44261 (11)	0.0188 (5)	
H13	0.6298	0.6902	0.4507	0.023*	

### Atomic displacement parameters (Å^2^)

**Table:**

	*U*^11^	*U*^22^	*U*^33^	*U*^12^	*U*^13^	*U*^23^
F1	0.0223 (9)	0.0372 (10)	0.0504 (11)	−0.0001 (7)	0.0114 (8)	−0.0089 (8)
F2	0.0312 (9)	0.0280 (9)	0.0430 (10)	0.0038 (8)	0.0066 (8)	−0.0127 (8)
N1	0.0202 (11)	0.0083 (9)	0.0236 (11)	0.0000 (8)	0.0013 (9)	−0.0010 (8)
N2	0.0216 (11)	0.0125 (10)	0.0204 (11)	0.0017 (9)	−0.0007 (9)	−0.0011 (8)
C1	0.0205 (13)	0.0122 (11)	0.0204 (13)	0.0040 (10)	−0.0022 (10)	0.0001 (10)
C2	0.0184 (12)	0.0117 (11)	0.0178 (12)	−0.0012 (10)	−0.0026 (10)	0.0017 (9)
C3	0.0179 (12)	0.0194 (13)	0.0261 (14)	0.0004 (11)	0.0063 (11)	0.0005 (11)
C4	0.0233 (13)	0.0178 (13)	0.0247 (14)	0.0055 (11)	0.0006 (11)	0.0001 (11)
C5	0.0245 (13)	0.0128 (12)	0.0240 (13)	0.0013 (10)	−0.0030 (11)	0.0002 (10)
C6	0.0185 (12)	0.0134 (12)	0.0205 (13)	−0.0027 (10)	−0.0031 (10)	0.0023 (10)
C7	0.0252 (14)	0.0113 (12)	0.0270 (14)	−0.0007 (10)	0.0031 (11)	0.0012 (10)
C8	0.0206 (13)	0.0146 (12)	0.0202 (13)	−0.0004 (10)	−0.0031 (10)	0.0047 (10)
C9	0.0278 (14)	0.0150 (12)	0.0257 (14)	−0.0007 (11)	0.0017 (11)	−0.0045 (11)
C10	0.0221 (14)	0.0246 (14)	0.0337 (15)	−0.0055 (12)	−0.0037 (12)	0.0003 (12)
C11	0.0164 (13)	0.0255 (14)	0.0281 (14)	0.0026 (11)	0.0041 (11)	0.0033 (11)
C12	0.0233 (13)	0.0134 (12)	0.0280 (15)	0.0041 (10)	−0.0010 (11)	−0.0011 (10)
C13	0.0183 (13)	0.0125 (12)	0.0257 (14)	−0.0008 (10)	−0.0002 (11)	0.0022 (10)

### Geometric parameters (Å, º)

**Table:**

F1—C11	1.344 (3)	C4—H4	0.9500
F2—C12	1.349 (3)	C5—C6	1.388 (4)
N1—C2	1.366 (3)	C5—H5	0.9500
N1—C1	1.378 (3)	C7—H7	0.9500
N1—H1	0.8800	C8—C9	1.386 (4)
N2—C2	1.326 (3)	C8—C13	1.386 (4)
N2—C6	1.393 (3)	C9—C10	1.396 (4)
C1—C7	1.394 (4)	C9—H9	0.9500
C1—C6	1.412 (3)	C10—C11	1.376 (4)
C2—C8	1.474 (4)	C10—H10	0.9500
C3—C7	1.388 (4)	C11—C12	1.379 (4)
C3—C4	1.403 (4)	C12—C13	1.371 (4)
C3—H3A	0.9500	C13—H13	0.9500
C4—C5	1.380 (4)		
			
C2—N1—C1	106.7 (2)	C3—C7—C1	117.1 (2)
C2—N1—H1	126.6	C3—C7—H7	121.5
C1—N1—H1	126.6	C1—C7—H7	121.5
C2—N2—C6	105.2 (2)	C9—C8—C13	119.5 (2)
N1—C1—C7	132.4 (2)	C9—C8—C2	118.9 (2)
N1—C1—C6	105.9 (2)	C13—C8—C2	121.6 (2)
C7—C1—C6	121.6 (2)	C8—C9—C10	121.4 (2)
N2—C2—N1	113.1 (2)	C8—C9—H9	119.3
N2—C2—C8	124.4 (2)	C10—C9—H9	119.3
N1—C2—C8	122.4 (2)	C11—C10—C9	118.0 (2)
C7—C3—C4	121.2 (2)	C11—C10—H10	121.0
C7—C3—H3A	119.4	C9—C10—H10	121.0
C4—C3—H3A	119.4	F1—C11—C10	119.8 (2)
C5—C4—C3	121.8 (2)	F1—C11—C12	119.5 (2)
C5—C4—H4	119.1	C10—C11—C12	120.6 (2)
C3—C4—H4	119.1	F2—C12—C13	120.9 (2)
C4—C5—C6	117.7 (2)	F2—C12—C11	117.6 (2)
C4—C5—H5	121.1	C13—C12—C11	121.4 (2)
C6—C5—H5	121.1	C12—C13—C8	119.1 (2)
C5—C6—N2	130.2 (2)	C12—C13—H13	120.5
C5—C6—C1	120.6 (2)	C8—C13—H13	120.5
N2—C6—C1	109.1 (2)		
			
C2—N1—C1—C7	176.2 (3)	C6—C1—C7—C3	0.7 (4)
C2—N1—C1—C6	−0.7 (3)	N2—C2—C8—C9	−26.6 (4)
C6—N2—C2—N1	−0.5 (3)	N1—C2—C8—C9	148.7 (2)
C6—N2—C2—C8	175.3 (2)	N2—C2—C8—C13	153.6 (2)
C1—N1—C2—N2	0.7 (3)	N1—C2—C8—C13	−31.1 (4)
C1—N1—C2—C8	−175.1 (2)	C13—C8—C9—C10	0.5 (4)
C7—C3—C4—C5	−0.3 (4)	C2—C8—C9—C10	−179.4 (2)
C3—C4—C5—C6	1.6 (4)	C8—C9—C10—C11	−0.8 (4)
C4—C5—C6—N2	175.1 (2)	C9—C10—C11—F1	−178.5 (2)
C4—C5—C6—C1	−1.6 (4)	C9—C10—C11—C12	0.4 (4)
C2—N2—C6—C5	−177.0 (3)	F1—C11—C12—F2	0.3 (4)
C2—N2—C6—C1	0.0 (3)	C10—C11—C12—F2	−178.5 (2)
N1—C1—C6—C5	177.8 (2)	F1—C11—C12—C13	179.2 (2)
C7—C1—C6—C5	0.5 (4)	C10—C11—C12—C13	0.4 (4)
N1—C1—C6—N2	0.4 (3)	F2—C12—C13—C8	178.2 (2)
C7—C1—C6—N2	−176.8 (2)	C11—C12—C13—C8	−0.7 (4)
C4—C3—C7—C1	−0.8 (4)	C9—C8—C13—C12	0.3 (4)
N1—C1—C7—C3	−175.7 (3)	C2—C8—C13—C12	−179.9 (2)

### Hydrogen-bond geometry (Å, º)

*Cg* is the centroid of the ring C9–C13 ring.

**Table:**

*D*—H···*A*	*D*—H	H···*A*	*D*···*A*	*D*—H···*A*
N1—H1···N2^i^	0.88	2.04	2.874 (3)	158
C13—H13···F2^ii^	0.95	2.51	3.379 (3)	153
C3—H3*A*···*Cg*^iii^	0.95	2.89	3.529 (3)	125

Symmetry codes: (i) −*x*+3/2, *y*+1/2, *z*; (ii) *x*+1/2, −*y*+3/2, −*z*+1; (iii) *x*+1/2, *y*, −*z*+1/2.
